# An Integrated System Combining Filter-Assisted Sample Preparation and Colorimetric Biosensing for Rapid Pathogen Detection in Complex Food Matrices

**DOI:** 10.3390/foods14172986

**Published:** 2025-08-27

**Authors:** Jihae Lee, Youngsang You

**Affiliations:** Department of Food Engineering, Dankook University, Cheonan 31116, Republic of Korea; jihae2019@dankook.ac.kr

**Keywords:** foodborne pathogens, complex food matrices, rapid diagnostics, filter-assisted sample preparation (FASP), colorimetric biosensor

## Abstract

Climate change increases microbial contamination risks in food, highlighting the need for real-time biosensors. However, food residues often interfere with detection signals, limiting the direct application. An integrated system of filter-assisted sample preparation (FASP) and an immunoassay-based colorimetric biosensor offers the rapid and simple on-site detection of foodborne pathogens in complex food matrices. The accuracy and stability of biosensor analysis were ensured via filter-assisted preprocessing, which separated food residues from bacteria. The system was applied to various food matrices, including vegetables, meats, and cheese brine, using samples spiked at contamination levels ranging from 10^2^ to 10^3^ CFU per 25 g, thereby demonstrating broad applicability. Bacterial recovery varied by food matrix, with vegetables showing a 1-log reduction and meats, melon, and cheese brine showing a 2-log reduction relative to the initial inoculum. A detection limit of 10^1^ CFU/mL was achieved for *Escherichia coli* O157:H7, *Salmonella* Typhimurium, and *Listeria monocytogenes* in the final preprocessed sample solutions. Sample preparation took under 3 min, and detection was completed within 2 h under stationary conditions. This approach enables rapid pathogen detection in various food matrices without the need for special reading devices, contributing to food safety as a real-time, rapid-response food biosensor.

## 1. Introduction

Foodborne pathogen outbreaks can occur worldwide at any stage of food production, processing, or storage, leading to significant financial losses and threats to public health [1,2]. According to the World Health Organization, unsafe food causes an estimated 600 million illnesses and 420,000 deaths each year, and in low- and middle-income countries, the total economic burden, including productivity losses and medical costs, is estimated to exceed US$ 110 billion [3,4]. However, gold-standard detection methods, including plate counts and PCR-based assays, require 24–72 h for culture-based enumeration and 4–6 h for PCR and demand specialized equipment and trained personnel, which limits their practicality for rapid foodborne pathogen detection [5,6]. To ensure food safety and comply with international trade regulations, the development of rapid pathogen diagnostic technologies applicable across the food industry is essential. Moreover, climate change is increasing the risk of microbial growth and contamination, further highlighting the need for food biosensors capable of real-time detection and a rapid response to environmental changes [7,8].

Food is a complex matrix containing proteins, fats, carbohydrates, and dietary fibers. Since these components can interfere with detection or reduce sensitivity, a preprocessing step to eliminate or mitigate their effects is essential [9,10]. In particular, fats, proteins, and pigments in food matrices can interfere with biosensor performance, reducing sensitivity or causing nonspecific signals that compromise detection accuracy [11,12]. According to ISO 6887-1:2017, solid food samples are typically homogenized using a stomacher to ensure uniform analysis [13]. Homogenization using a stomacher applies mechanical force to liquefy solid food samples and extract pathogens, playing a crucial role in reliable detection [14]. However, residual food components can interfere with biosensors through nonspecific interactions that affect detection sensitivity [15], making it particularly challenging to detect low levels of pathogens [16]. Moreover, the lack of a rapid, universal preprocessing method remains a barrier to on-site rapid diagnostics. Several studies have attempted to overcome these limitations by designing preprocessing protocols tailored to specific food matrices or by incorporating filtration and separation techniques [17,18]. However, their applicability across diverse and complex food matrices remains uncertain due to variations in composition. Even conventional methods such as PCR require matrix-specific and optimized sample preparation, as inhibitors inherent to complex food matrices can compromise template integrity and amplification efficiency, potentially resulting in false negatives or reduced sensitivity [19,20]. For example, detection limits for pathogens using TaqMan PCR were about one log higher in real meat samples than in pure cultures [21].

Research on biosensors has predominantly focused on detecting pathogens in simplified or purified sample conditions, limiting their applicability in real-world food matrices [22]. For example, in complex matrices such as milk, an AuNP-based LFA detected 8.6 × 10^0^ CFU/mL in pure *Salmonella* cultures, but the LOD increased to 4.1 × 10^2^ CFU/mL, highlighting the challenges of complex food matrices based on biosensor performance [23], as observed in other studies as well [24]. Among various biosensor platforms, electrochemical biosensors, while offering high sensitivity and portability, are particularly susceptible to matrix-induced interference [25]. Other formats, such as lateral flow assays (LFAs), surface plasmon resonance (SPR), and quartz crystal microbalance (QCM), also require extensive preprocessing procedures, including centrifugation, filtration, or buffer adjustment to mitigate matrix effects [26,27,28]. Table 1 provides a summary of representative rapid detection strategies, outlining their detection principles, sample types, preprocessing requirements, and detection limits. Techniques such as centrifugation, immunomagnetic separation (IMS), and filtration face limitations related to sensitivity, field applicability, the complexity of preprocessing, and dependence on specialized equipment. Although some systems have been tested with real samples, broader and more systematic validation across diverse food matrices is still required to ensure reliable real-time pathogen detection.

Recent approaches to improve the applicability of biosensors in real food matrices have focused on integrating more efficient preprocessing techniques [29,30]. Previous studies have employed strategies such as double filtration systems, combining a primary filter to remove large food particles and a secondary filter to capture target bacteria, in order to simplify sample preparation and enhance target recovery [31]. Building on this approach, *Escherichia coli* O157:H7 contamination at a level of 10^2^ CFU in 25 g of tomato was successfully detected using an immunoassay-based colorimetric biosensor. These results demonstrate that controlling matrix-derived interfering substances in food can reduce nonspecific reactions, thereby improving the accuracy and reliability of the biosensor. Another study employed a single filtration method for vegetable samples [32], whereas a dual-filtration system utilizing filters with different pore sizes first removes larger particles and subsequently captures microorganisms in the secondary filter. Thereby facilitating more consistent processing in complex food matrices. In addition, specific antigen–antibody interactions were used to reduce interference from complex food matrices, while the optical properties of nanoparticles enable direct visual detection without extensive sample purification, making the system simple, specific, and suitable for field applications [11,33]. Therefore, this study aims to develop an integrated detection system that combines simple, filter-assisted sample preparation with an immunoassay-based colorimetric biosensor to overcome the complexity of diverse food matrices. Target bacteria can be detected at 10^1^ CFU/mL without enrichment or amplification, with visual analysis completed within two hours. To validate its practical applicability, the system was applied to a wide range of food matrices with diverse physicochemical properties, including vegetables, meats, and dairy-derived samples, demonstrating broad applicability and robustness across these sample types. Unlike previous studies that often required complex preprocessing or specialized equipment, the proposed approach highlights the simplicity, sensitivity, and potential for on-site food safety monitoring, enabling pathogen detection directly across complex matrices with minimal handling and instrumentation.

**Table 1:** Summary of previous studies and comparison with this work.

Preprocessing Method	Detection Method	Instrument	Preprocessing/ Detection Time	LOD	Sample	Target	Reference
Double filter method: GF/D and cellulose acetate filter with 0.45 μm-sized pore	Immunoassay-based colorimetric biosensor	Stomacher Vacuum pump	≥3 min/ ≥120 min	10^1^ CFU/mL	Vegetables Meats Cheese brine, etc.	*E. coli* O157:H7 *S.* Typhimurium *L. monocytogenes*	This work
Immunomagnetic separation	Aptamer-based QCM sensor	Centrifuge QCM crystal Homogenizer Frequency counter Magnetic separator	≥10 min/ ≥5 min	10^2^ CFU/mL	Poultry Milk	*L. monocytogenes*	Beyazit et al., 2024 [34]
Filter method: Paper filter and centrifugation method	Nanozyme-based colorimetric biosensor	Centrifuge Paper-chip	N.D.^(1)^ ≥120 min	10^1^ CFU/mL	Milk	*S. Typhimurium*	Mirsadoughi et al., 2023 [35]
Pre-enrichment	Immunoassay-based optical biosensor	Incubator Photodetector Microfluidic system Nanophotonic biosensor	≥Total 4 h	10^1–2^ CFU/mL	Hamburger patty	*L. monocytogenes*	Blanco et al., 2023 [36]
N.D.	DNA-based Ectrochemical biosensor	Vibrator Centrifuge Heating source Electrochemical instrument	N.D.	10^0^ CFU/mL	Egg Raw milk Poultry Human blood	*S*. Typhi	Bacchu et al., 2022 [37]
Filter method: glass wool, graphite electrode, and filter with 50 μm-sized pore, continuous flow centrifuge	Enzyme-linked immunoelectrochemical biosensor	Stomacher Vacuum pump Continuous flow centrifuge Electrochemical instrument	N.D. ≥3 h	10^2^ CFU/mL	Minced beef	*E. coli* O157:H7	Capobianco et al., 2021 [38]
Centrifugation method Pre-enrichment	Immunoassay-based multistep lateral flow assay	Stomacher Centrifuge Incubator LFlIA strips	≥Total 7 h	10^0^ CFU/g	Lettuces	*E. coli* O157:H7 *S.* Typhimurium *S. aureus* *B. cereus*	Shin et al., 2018 [39]

^(1)^ N.D. indicates that no specific information was provided in the corresponding study.

## 2. Materials and Methods

### 2.1. Filter-Assisted Sample Preparation

#### 2.1.1. Food Samples

The real matrices chosen for testing included vegetables, such as cabbage (E-Mart partner farm, Cheonan, Republic of Korea), carrot (Daehan Nongsan, Busan, Republic of Korea), cucumber (Hwashin Nongsan, Gwangju, Republic of Korea), romaine lettuce (Onchae Agricultural Cooperative, Nonsan, Republic of Korea), and melon (Seji Agricultural Cooperative, Naju, Republic of Korea); meats including chicken (Harim, Iksan, Republic of Korea), pork (Myungjin MS, Jincheon-gun, Republic of Korea), and beef (Mirae Wellfood, Seongnam, Republic of Korea); egg shell (Daon & Farm, Cheonan, Republic of Korea); and the brine from soft cheese (Dairygen, Wonju, Republic of Korea). All samples were purchased from local supermarkets, stored at 4 °C, and used within 24 h of purchase to maintain freshness and minimize changes in microbial survival and surface conditions. Each sample was analyzed through plate counting after the filtration process to confirm the absence of target bacteria. Prior to filtration, 25 g of each sample was prepared as a single piece to the extent possible, with cheese brine, being a liquid sample, used as is without further processing. The thickness and size of the samples varied depending on their physical characteristics.

#### 2.1.2. Filtration Process

**Figure 1:**
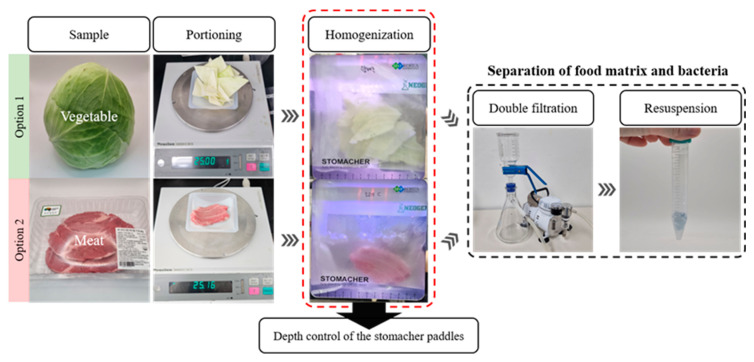
Overview of filter-assisted sample preparation strategies based on sample matrices. Samples were homogenized in 0.85% NaCl, vacuum-filtered through GF/D and CA membranes, and bacteria were recovered from the secondary filter. To minimize filtration delays, the operating depth of the stomacher paddles was adjusted for meat samples (e.g., beef) to reduce fine particulate release. Other steps, including sampling, filtration, and secondary filter recovery, were consistent across all samples.

The filtration process, illustrated in Figure 1, was based on the method developed by Han et al. [31], with slight modifications to the homogenization step depending on the characteristics of each sample matrix. Each sample was wiped with Kimtech tissue and 70% (*v*/*v*) ethanol (Samchun Pure Chemical, Pyeongtaek, Republic of Korea) to remove surface contaminants, and residual ethanol was evaporated at room temperature for 15 min. Then, 25 g of the sample was homogenized with 225 mL of 0.85% (*w*/*w*) NaCl for 2 min using a stomacher (LS-400, BNF Korea, Bucheon, Republic of Korea). For certain samples, such as poultry meat, which tends to release fine particulates likely to clog filter pores, the operating depth of the stomacher paddles was reduced to limit the release of solids during homogenization. This adjustment enabled the filtration speed to be aligned with that of vegetable samples reported in the study by Han et al. [31], where the primary and secondary filtrations were completed within approximately 10 s and 1 min, respectively. Detailed stomacher settings for each sample matrix are summarized in Table S1. The homogenate was primary filtered through a 2.7 μm-pore-size GF/D filter (Whatman, Maidstone, UK), followed by secondary filtration through a 0.45 μm-pore-size CA filter (Advantec, Saitama, Japan). In brief, the primary filter removes large particulate from the homogenized food sample, while the secondary filter captures the target bacteria in the solution. The secondary filter was resuspended in 2 mL of 0.85% NaCl by vortexing (US-VM, WooJu Science, Guri, Republic of Korea) three times for 3 s each. Each filter was not pre-wetted before filtration, and the primary and secondary filtrations typically took approximately 10 s and 1 min, respectively. The filtration process was carried out under pressure using a vacuum pump (LAB300, Lab Touch, Seoul, Republic of Korea) with a maximum pressure of 690 mmHg and a maximum vacuum of 920 mbar.

#### 2.1.3. Recovery of Bacteria from Various Food Matrices

To evaluate the bacterial recovery in different food matrices, 10^3^ CFU of *Escherichia coli* O157:H7 was inoculated onto 25 g of cabbage, carrot, cucumber, and romaine lettuce, while 10^3^ CFU of *Salmonella* Typhimurium was inoculated onto 25 g of chicken, pork, and beef. Additionally, 10^3^ CFU of *Listeria monocytogenes* was inoculated onto 25 g of melon and cheese brine. In the case of melons, samples were taken with the rind intact, and inoculation was performed on the rind. The inoculated samples were spot-inoculated with 100 µL of bacterial suspension and incubated at room temperature for 15 min to facilitate bacterial attachment to the food surface. The samples then underwent both primary and secondary filtration steps, as described in Section 2.1.2, after which the bacteria concentrated on the secondary filter were resuspended to prepare a preprocessed food sample solution. Subsequently, bacterial recovery was evaluated by plating onto selective media specific to each pathogen.

### 2.2. Colorimetric Biosensing

#### 2.2.1. Biosensor Materials

Gold (III) chloride trihydrate (HAuCl4·3H_2_O), streptavidin (from Streptomyces avidini), and Tri-sodium citrate dihydrate were purchased from Sigma-Aldrich (St. Louis, MO, USA). Biotin-labeled *Escherichia coli* antibody, *Salmonella* spp. group antibody, and *Listeria* sp. group antibody were obtained from Thermo Fisher Scientific (Waltham, MA, USA). Bovin serum albumin was purchased from HanLAB (Cheongju, Republic of Korea).

#### 2.2.2. Synthesis of Streptavidin-Functionalized Gold Nanoparticles (AuNPs)

Aqueous gold nanoparticles (AuNPs) with a diameter of 13 nm were synthesized by reducing HAuCl_4_·3H_2_O aqueous solution with sodium citrate at its boiling point, following a published procedure [40,41]. The colloidal AuNP solution exhibited an absorbance of 0.4 at a wavelength of 520 ± 0.5 nm. The size and distribution of the AuNPs were characterized using a UV–vis spectrophotometer (G1103A, Agilent Technologies, Santa Clara, CA, USA) and through dynamic light scattering (ZMV2000, Malvern Panalytical, Malvern, UK), respectively.

AuNPs were coated with streptavidin according to a published method [42,43]; 600 µL of colloidal AuNPs was mixed with 200 µL of streptavidin (0.2 mg/mL in borate buffer). Excess streptavidin was removed via centrifugation at 10,000 rpm for 30 min using an Eppendorf 5415 C centrifuge (Eppendorf SE, Hamburg, Germany). The pellet was resuspended in 0.1% (*w*/*v*) BSA in phosphate buffer saline (PBS). And the streptavidin-functionalized AuNPs (st-AuNPs) were adjusted to an absorbance of 0.2 at 530 ± 0.5 nm.

#### 2.2.3. Immunoassay-Based Colorimetric Biosensor

**Figure 2:**
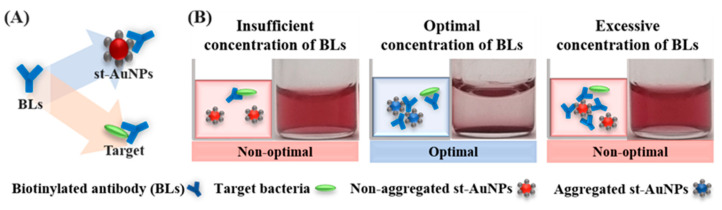
Colorimetric immunoassay-based colorimetric biosensor: the role of bi-functional linkers. (**A**) The BLs (bi-functional linker) can bind either to the streptavidin functionalized gold nanoparticles (st-AuNPs) or to the target. (**B**) This leads to the large-scale aggregation of st-AuNPs at an optimal linker concentration, producing a visible color change.

The biosensor system utilizes streptavidin-coated AuNPs (st-AuNPs) and biotin-labeled bacterial antibodies (b-Abs) as bi-functional linkers (BLs). The process begins with an antibody–antigen immune reaction, where the BLs (biotinylated antibodies) specifically bind to the target antigen or interact with st-AuNPs via biotin–streptavidin binding to induce a colorimetric change (Figure 2A). A specific concentration of BLs induces sufficient aggregation, resulting in a color change, referred to as the range of visible color change (REVC) [42]. Depending on the BL concentration, aggregation shows three regions: insufficient (no color change), optimal (visible aggregation), and excessive (aggregation inhibited) (Figure 2B). The system also includes a control sample without the target to validate the detection process. The total reaction volume of 400 µL contained 200 µL of st-AuNPs, 100 µL of BLs, and 100 µL of preprocessed sample solution. After incubating for about 1 h to allow the antibody–antigen reaction, st-AuNPs were added to the mixture and incubated for an additional 2 h under stationary conditions. Visual detection was considered positive when the REVC, indicated by a color change from red to pale, shifted rightward compared with the control (target-free). Additionally, the aggregation and precipitation of st-AuNPs shifted the λ_max_ to longer wavelengths, and the REVC region defined based on λ_max_ was considered positive when it shifted rightward in the presence of the target.

### 2.3. Impact of Sample Preparation on Biosensing Performance

The immunoassay system’s functionality was first evaluated in PBS buffer, following the procedure described in Section 2.2.3. A rightward shift in REVC and a change in λ_max_ served as indicators of the presence of target bacteria. Negative and positive groups of *Escherichia coli* O157:H7, *Salmonella* typhimurium, and *Listeria monocytogenes* were included for validation.

To verify the compatibility of the filter-assisted sample preparation with the biosensor, 25 g of food samples was prepared using four sample-preparation methods: the developed preprocessing method (Section 2.1.2), blending, stomacher homogenization, and primary filtration. For blending, 25 g of the sample was mixed with 225 mL of solution and finely ground using a blender. According to the standard food microbiological test method, stomacher homogenization was performed by processing 25 g of the sample with 225 mL of solution for 2 min. For the filtration method, the test solution was obtained by collecting the primary filtrate following stomacher homogenization. Each preprocessed sample solution was used as the target (100 µL) for the biosensor according to the method of Section 2.2.3.

### 2.4. Application of the Integrated Diagnostic System in Various Food Matrices

To evaluate detection efficiency in real food matrices, 25 g of cabbage, carrot, cucumber, and romaine lettuce was inoculated with *Escherichia coli* O157:H7 (10^2^ CFU), 25 g of chicken, pork, and beef was inoculated with *Salmonella* Typhimurium (10^3^ CFU), and 25 g of melon and cheese brine was inoculated with *Listeria monocytogenes* (10^3^ CFU). The preprocessed food sample solutions were prepared as described in Section 2.1.2. As described in Section 2.2.3, detection was performed using the colorimetric biosensor. First, 100 μL of each biotinylated antibody solution (0, 1, 5, 10, 15, and 25 μg/mL) was mixed with 100 μL of the pretreated sample solution. The mixture was gently stirred every 15 min for 1 h to facilitate the antigen–antibody reaction. Then, 200 μL of st-AuNPs (wavelength: 530.0 ± 0.5 nm, absorbance: 0.2) was added, and the reaction was allowed to proceed for an additional 2 h. The detection results were confirmed visually and using a UV–visible spectrophotometer.

### 2.5. Bacteria Culture

The target bacterial strains used were *Escherichia coli* O157:H7 43895, *Salmonella* typhimurium DT-104, and *Listeria monocytogenes* ATCC 19115. The bacterial cultures were grown for 12–24 h in Tryptic Soy Broth (TSB) at 37 °C. The cultures were centrifuged at 4000 RPM (3243× *g*) for 30 min, and the resulting pellets were resuspended in sterile PBS. Each bacterial suspension at 10^9^ CFU/mL was diluted in PBS to obtain the desired inoculum concentration, and each dilution was verified via plate counting.

## 3. Results and Discussion

### 3.1. Bacterial Recovery from Various Food Matrices

Pathogenic *Salmonella* spp. and *Escherichia coli* are recognized as major contaminants in meat, eggs, and vegetables, respectively, and represent significant causes of foodborne illnesses [44,45]. In addition, *Listeria monocytogenes*, a psychrotrophic bacterium capable of surviving and proliferating at refrigeration temperatures, is frequently found in chilled products such as melons and dairy items, posing a critical risk in cold chain food safety management [46,47]. However, despite the importance of detecting these pathogens, accurate analysis remains challenging due to food matrix effects [48]. Food matrices contain components such as carbohydrates, lipids, and proteins, which can interfere with microbial detection by increasing sample viscosity, inhibiting nucleic acid amplification, or disrupting antigen–antibody interactions. For example, high fat and protein in meat and dairy can hinder DNA extraction and enzymatic reactions, while polysaccharides and phenolics in vegetables can reduce the sensitivity of immunoassays and colorimetric biosensors [49,50]. Therefore, appropriate sample preprocessing is essential to eliminate such matrix effects and to obtain accurate and reproducible analytical results.

Table 2 summarizes the changes in target bacterial concentrations observed during the filter-assisted sample preprocessing. The photographs of target bacteria recovered from the secondary filter and cultured on selective media are shown in Figure S1. No target bacteria were detected in any samples prior to spiking or in the filtrates obtained after secondary filtration. Although the filtration speed varied depending on the type of food matrix, the average processing time was approximately 10 s for the primary filter and about 1 min for the secondary filter. In particular, samples such as poultry and other meat products tended to release fine particulates during homogenization, which could clog filter pores and the filtration process. To address this issue, the penetration depth of the stomacher paddles was adjusted in meat samples to limit the release of solid particles. The penetration depth refers to the depth to which the paddles press into the sample, influencing the degree of tissue disruption and the amount of particulates released during homogenization. The stomacher condition used for vegetable samples, including cabbage, carrot, cucumber, romaine lettuce, and melon consisted of a penetration depth of 15 mm at level 7 for 2 min. For meat-based samples such as chicken, pork, and beef, only the paddle depth was adjusted to 5 mm, while the speed and duration remained constant. This adjustment enabled the filtration process for meat samples to be completed within approximately 10 s for the primary and 1 min for the secondary filters, respectively, which was comparable to the preprocessing times observed in previous studies using vegetable matrices. Adjusting the penetration depth of the stomacher paddles did not result in a significant difference in bacterial recovery on a logarithmic scale (Table S2). Although melon followed the same homogenization protocol as the vegetables, its high sugar content and the release of fine cellular debris occasionally increased sample viscosity, leading to minor delays [51,52]. For cheese brine, a liquid sample, homogenization was not performed, as no liquefaction step was required.

Each food sample was inoculated with 10^3^ CFU/25 g of the target bacteria, and a final volume of 2 mL of the preprocessed sample solution was plated on selective media to determine the bacterial recovery rate. CFU/total refers to the total number of colony-forming units recovered from the entire resuspended volume, whereas CFU/mL indicates the bacterial concentration per milliliter of that volume. Spiked samples refer to solutions obtained by inoculating 25 g of sample, followed by a 10-fold dilution and homogenization with a stomacher, according to standard microbiological procedures. Compared to the FASP developed in this study, the conventional stomacher method often results in lower recovery efficiency and higher variability. Small-volume sampling and a 10-fold dilution step can lead to reduced recovery and sensitivity. As a result, the bacterial concentration per mL is lower than that obtained using the FASP method.

According to the study by Han et al. [31], the preprocessing method yielded approximately 10^1^ CFU/mL of sample solution from an initial inoculum of 10^2^ CFU per 25 g of tomato. Comparable levels of bacterial reduction were observed in other vegetables, including cabbage, carrot, cucumber, and romaine lettuce. The total number of bacteria retained on the secondary filter from vegetable samples inoculated with 10^3^ CFU per 25 g was approximately 10^2^ CFU, indicating a 1-log reduction during the filtration process. Dietary fiber, a major component of vegetable matrices, is predominantly insoluble and does not produce fine particulates capable of clogging filter pores, even after mechanical disruption such as stomacher homogenization [53]. As a result, filtration proceeds efficiently, and microbial recovery is significantly high in matrices such as meat, which typically exhibit greater viscosity and particulate content [54]. However, soft matrices such as melons produce insoluble particulates during homogenization that clog filters and increase viscosity [55,56]. Though high sugar can contribute, insoluble debris likely plays a more significant role due to the physical nature of filtration, causing pretreatment delays and reduced recovery. In melon samples inoculated with 10^3^ CFU per 25 g, approximately 10^1^ CFU remained on the secondary filter, reflecting a 2-log reduction during filtration.

For poultry and meat samples, bacterial concentrations in the resuspended solution from the secondary filter were reduced by approximately 2-log compared to the initial inoculum (per 25 g). This reduction can be attributed to the relatively high protein and fat contents of meat, which can enhance bacterial adhesion to filter surfaces or matrix particulates [57,58]. In addition, meat samples often exhibit viscosity changes depending on freshness and contain fine particulates, such as muscle fibers, that can prolong preprocessing and reduce bacterial recovery [59,60]. As a result, the recovery rate was lower than that observed for vegetable samples. Eggshells are a major contamination site for *Salmonella*, as pathogens can remain on the surface through contact with the external environment during laying and distribution [61]. While the egg contents are typically sterilized by cooking, the shell poses a higher risk of cross-contamination, making it an important target for contamination monitoring [62,63]. However, in the case of eggshells, high-viscosity proteins such as albumin led to severe gelation or membrane fouling on the filter surface [64], effectively halting the filtration process and rendering filter-assisted pretreatment infeasible in practice (Table 2).

This study demonstrates the broad applicability for rapid pathogen detection across various food matrices by minimizing matrix interference. However, components such as high viscosity, fats, and solid particulates in complex food matrices can reduce filtration efficiency, necessitating the optimization of bacterial capture and resuspension, potentially through chemical dissolution methods. Despite these challenges, the system exhibited high sensitivity and reproducibility, supporting its potential for reliable on-site detection. Based on bacterial recovery patterns in various food matrices, achieving approximately 10^1^ CFU/mL in the final sample solution for sensor application required different initial inoculum levels depending on the matrix. For vegetable samples, about 10^2^ CFU per 25 g was needed, whereas for meat, melon, and cheese brine samples, approximately 10^3^ CFU per 25 g was necessary.

### 3.2. Influence of Sample Preparation on Biosensing Outcomes

**Figure 3:**
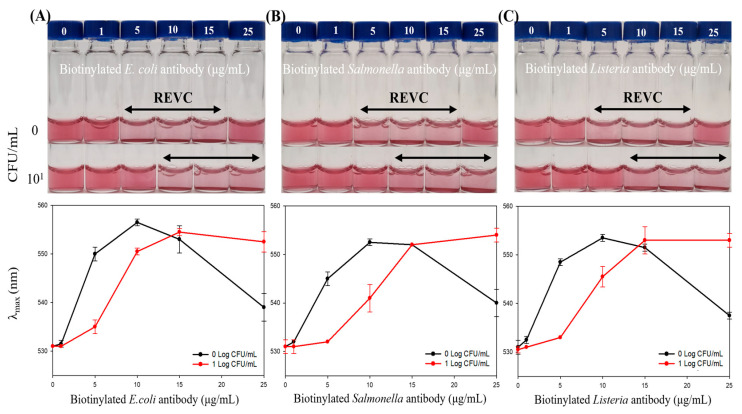
Detection of target bacteria in PBS buffer. Colorimetric responses and shifts in λ_max_ were evaluated via visual observations and spectrophotometry. (**A**) *Escherichia coli* O157:H7. (**B**) *Salmonella* Typhimurium. (**C**) *Listeria monocytogenes*.

The immunoassay system was first evaluated in PBS buffer to confirm its detection capability. When target bacteria were present at 10^1^ CFU/mL, a rightward shift in the REVC range (from 10–15 μg/mL to 15–25 μg/mL) and a change in λ_max_ were observed (Figure 3). Both the visible detection range and λ_max_ shifted right, enabling target detection by the naked eye and via spectrophotometry. These findings indicate that the system functions as an on–off platform to distinguish the presence or absence of target bacteria. The limit of detection (LOD) was determined to be 10^1^ CFU/mL for *Escherichia coli* O157:H7, *Salmonella* Typhimurium, and *Listeria monocytogenes* in PBS.

**Figure 4:**
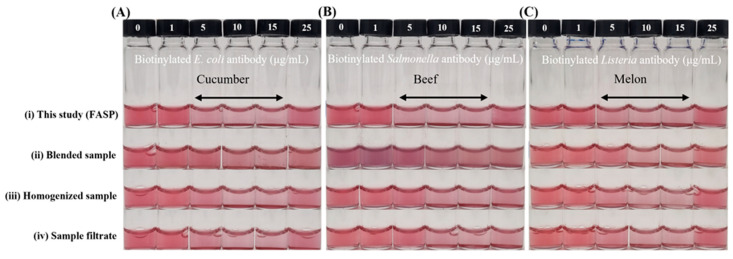
Importance of integrating food sample preparation with colorimetric biosensing. Colorimetric biosensor integrated with various pretreatment methods for (**A**) cucumber, (**B**) beef, and (**C**) melon. From top to bottom: (i) method developed in this study, (ii) blended sample, (iii) homogenized sample, (iv) sample filtrate.

Various sample preparation methods integrated with a colorimetric biosensor were visually compared to the filter-assisted preprocessing method developed in this study, using complex food matrices such as beef, cucumber, and melon (Figure 4). This biosensor system required a negative control (target-free sample) without the target for detection, as a stable signal from the target-free control was essential to reliably distinguish the presence of the target. The integrated system developed in this study demonstrated superior stability, accuracy, and reproducibility across these diverse matrices. In contrast, samples prepared by blending, stomacher homogenization, or primary filtration exhibited nonspecific interference in negative control samples, with signals tending to shift right-ward, similar to those of positive controls (Figure S2). In some cases, excessive color changes caused by nonspecific aggregation interfered with sensor performance. Moreover, these nonspecific responses varied unpredictably between sample types, leading to instability in the negative controls and leading to difficulty in distinguishing target presence. Especially, in the case of the blended beef sample, the strong influence of the sample matrix, including its inherent color and interfering substances, caused the excessive nonspecific aggregation of gold nanoparticles, making identification using the sensor completely impossible (Figure 4B). By comparison, the integrated system produced negative control signals that closely resembled those observed in PBS buffer, forming control REVC values in the range of 10–15 μg/mL, indicating the effective suppression of matrix-derived interference and reliable performance across diverse food samples.

Inherent background signals in complex food matrices pose significant challenges to biosensor accuracy and reproducibility. Colorimetric signal-generating st-AuNPs are generally stable due to electrostatic and steric effects. However, complex food matrices can disrupt this stability and induce aggregation. Near streptavidin’s isoelectric point (pH 5–6), electrostatic repulsion is weakened, promoting aggregation [65]. Additionally, high ionic strength compresses the electrical double layer, increases van der Waals interactions, and destabilizes nanoparticles [66]. These phenomena can be influenced by the food matrix’s physicochemical properties, notably its broad pH range and varying ionic strength. Furthermore, in foods such as meat, abundant proteins and lipids facilitate nonspecific adsorption and hydrophobic interactions [67,68]. These variations in pH, ionic strength, and nonspecific matrix components can significantly alter negative control signals and target-induced color shifts resulting in inconsistent and unreliable detection [69,70]. Collectively, these results underscore the need for effective pretreatment to remove inherent food interferences, maintain nanoparticle stability, and ensure reliable biosensor performance.

### 3.3. Performance of the Integrated Diagnostic System in Various Food Matrices

Detecting pathogens in complex food matrices poses significant challenges due to their diverse physicochemical properties, which interfere with target recognition and nanoparticle aggregation. For example, without pretreatment, the limit of detection (LOD) in whole milk was reported to be higher (10^2^ CFU/mL) than in simpler matrices such as PBS (10^1^ CFU/mL) [71]. To overcome matrix interference and assess the practical applicability of this study across various matrices, real samples were pretreated using FASP, and the resulting changes in the REVC range were analyzed.

**Figure 5:**
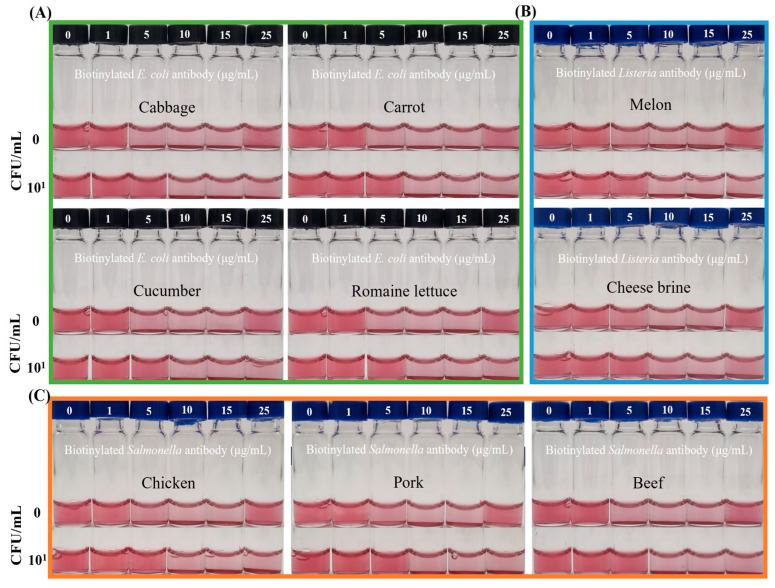
Detection of foodborne pathogens in various food matrices. (**A**) Detection of *Escherichia coli* O157:H7 in cabbage, carrot, cucumber, and romaine lettuce. (**B**) Detection of *Salmonella* Typhimurium in chicken, pork, and beef. (**C**) Detection of *Listeria monocytogenes* in melon and cheese brine. In the data for each sample, the photo above is a food sample control, and the photo below is a food sample inoculated with target bacteria. With the presence of 10^1^ CFU/mL of target bacteria in preprocessed sample solutions, the REVC shifted to the right.

The system was evaluated using spiked samples at contamination levels of 10^2^–10^3^ CFU/25 g, with each food type tested alongside a target-free negative control. Simple preprocessed real food samples, including vegetables such as cabbage, carrot, cucumber, romaine lettuce, and melon; meats including chicken, pork, and beef; and the brine from soft cheese performed comparably to PBS in the detection assay (Figure 5). Each sample showed minor variations in the REVC, system color, and UV–vis data (Figure S3), likely due to the intrinsic components present in the food matrices. However, these differences were negligible, as consistent and reliable detection was achieved when target bacteria were inoculated and tested, similar to results obtained with PBS buffer. This stability indicates that the sample preparation method developed in this study effectively minimized matrix-derived interference from complex food samples.

When tested with target pathogens at 10^1^ CFU/mL in the preprocessed sample solutions, the REVC shifted rightward from 5–10 to 10–25 μg/mL, consistent with results obtained using PBS buffer. This demonstrates that the streptavidin–biotin interaction, which is critical for visible signaling, remained effective even in complex food matrices. Combined with colorimetric biosensing, this integrated approach enabled the sensitive detection of pathogens within 2 h, achieving a limit of detection (LOD) of 10^1^ CFU/mL for *Escherichia coli* O157:H7, *Salmonella* Typhimurium, and *Listeria monocytogenes*.

In this study, a simple filter-assisted sample preparation (FASP) was applied directly to various food samples without pre-enrichment or amplification. The FASP procedure, completed within 3 min, maintained speed and reproducibility across various matrices. Coupled with a colorimetric biosensor, it enabled foodborne pathogen detection within 2 h, achieving a detection limit of 10^1^ CFU per mL of the preprocessed food sample. The detection time under static conditions could be further reduced by miniaturizing and integrating mixing, reaction, and precipitation steps, as well as applying physical forces (e.g., shaking). Its simplicity and reliable sensitivity make it suitable for portable, on-site food safety applications.

## 4. Conclusions

This study demonstrated that a rapid and simple filter-assisted sample preparation method, completed within 3 min, effectively mitigated matrix interferences across various food samples, enabling visual detection through an immunoassay-based colorimetric biosensor. Simple preprocessing removed food residues: the primary filter eliminated large particles, while the secondary filter captured pathogens, thereby enhancing target recovery and reducing matrix effects. This streamlined method enabled the on/off detection of as low as 10^1^ CFU/mL of *Escherichia coli* O157:H7, *Salmonella* Typhimurium, and *Listeria monocytogenes* within 2 h using an immunoassay-based colorimetric biosensor. An integrated system, combining filter-assisted sample preparation with colorimetric biosensing, was successfully applied to various food matrices, including vegetables (cabbage, carrot, cucumber, romaine lettuce), meats (chicken, pork, beef), and other food products such as melon and cheese brine.

Differences in recovery were observed depending on the specific characteristics of each food matrix: vegetables showed approximately a 1-log reduction compared to the inoculated level, while meats, melon, and cheese brine showed a trend of about a 2-log reduction. However, under the experimental conditions used in this study, the applicability of this system can be limited in complex food matrices, such as eggs, where high viscosity impairs filtration efficiency. In particular, the high viscosity of protein-rich eggs leads to membrane fouling and reduced flux, directly hindering the filtration of extracts containing viable microbial cells [72]. Future research should develop advanced pretreatment strategies, such as enzymes, ionic liquids, surfactants, or lysis buffers, applied during sample homogenization to minimize interference with downstream detection, for example, by using proteolytic enzymes that hydrolyze egg proteins without affecting the viability of target microbial cells. These approaches aim to enhance microbial recovery while maintaining nanoparticle stability in high-fat and protein-rich food samples, which impede filtration efficiency and compromise pathogen release and detection sensitivity. These refinements are essential to expand the platform’s applicability to matrices with complex physicochemical and structural characteristics.

Nevertheless, these findings suggest that the developed system offers significant advantages, including high reproducibility across divers matrices and enhanced field applicability by minimizing matrix interference. Its simplicity, portability, and detection capability position it as a promising tool for on-site food safety monitoring, particularly in the context of increasing climate-driven contamination risks.

## Figures and Tables

**Table 2:** Microbial concentration in filter-assisted sample preparation from various food matrices.

Target (10^3^ CFU)	Sample (25 g)	Concentration of Bacteria (CFU/Total)	Concentration of Bacteria (CFU/mL)			
		Resuspended Second Filter ^(1)^	Control ^(2)^	Spiked Sample ^(3)^	Resuspended Second Filter	Estimated log Reduction
*Escherichia coli* O157:H7	Romaine lettuce	163.7 ± 8.2 ^(4)^	ND ^(5)^	4.6 ± 0.5	81.8 ± 4.1	1-log reduction
	Cabbage	208.3 ± 37.5	ND	5.6 ± 1.2	104.1 ± 18.8	
	Cucumber	205.0 ± 16.4	ND	6.6 ± 1.2	102.5 ± 8.2	
	Carrot	181.0 ± 19.1	ND	5.3 ± 0.9	90.5 ± 9.6	
*Salmonella* Typhimurium	Chicken	47.3 ± 8.2	ND	11.0 ± 3.0	23.7 ± 4.1	2-log reduction
	Pork	53.6 ± 21.7	ND	12.0 ± 3.3	26.8 ± 10.8	
	Beef	31.7 ± 7.4	ND	11.0 ± 2.2	15.8 ± 3.7	
	Egg shell	N/A ^(5)^	ND	6.6 ± 0.9	N/A	N/A
*Listeria monocytogenes*	Melon	122.7 ± 38.0	ND	8.7 ± 2.6	61.3 ± 19.0	2-log reduction
	Cheese brine	132.7 ± 11.0	ND	7.0 ± 1.6	66.3 ± 5.5	

^(1)^   Secondary filtrate obtained after double filtration of the inoculated sample using the FASP method; ^(2)^ control: non-inoculated sample, diluted (1:10) and homogenized using a stomacher; ^(3)^ spiked samples: s amples inoculated, diluted (1:10), and homogenized using a stomacher; ^(4)^ data were measured at least three times and are presented as the mean ± SD; ^(5)^ ND means not detected; N/A means not applicable due to experimental limitations.

## Data Availability

The original contributions presented in this study are included in the article and Supplementary Material. Further inquiries can be directed to the corresponding author.

## Supplementary Materials

The following supporting information can be downloaded at: https://www.mdpi.com/article/10.3390/foods14172986/s1, Table S1: Stomacher homogenization conditions used for each sample matrix.; Table S2: Effect of the stomacher paddle penetration depth on bacterial recovery in chicken.; Figure S1: Plating results on selective media after filter-assisted sample preparation of real food samples.; Figure S2: Changes in λ_max_ of colorimetric biosensors integrated with various pretreatment methods, as measured via spectrophotometry.; Figure S3: Variation in λ_max_ in response to foodborne pathogen detection in various food matrices using an immunoassay-based colorimetric biosensor.
