# Supplementary material for: An Integrated System Combining Filter-Assisted Sample Preparation and Colorimetric Biosensing for Rapid Pathogen Detection in Complex Food Matrices
Source: Foods. 2025 Aug 27;14(17):2986. doi: 10.3390/foods14172986 (PMC12428675; doi:10.3390/foods14172986)
Supplement: Supplementary file 1 [file foods-14-02986-s001.zip › foods-3818850-supplementary.pdf]

Table S1. Stomacher homogenization conditions used for each sample matrix.

Sample	Distance (depth)	Level (strength)	Time
Romaine lettuce<br>Cabbage<br>Cucumber<br>Carrot	15 mm	7	2 min
Chicken<br>Pork<br>Beef<br>Carrot	5 mm	7	2 min
Egg shell	15 mm	7	2 min
Cheese brine		N/A[1)]	

1) N/A: Liquid samples were filtered without stomacher homogenization.

Table S2. Effect of the stomacher paddle penetration depth on bacterial recovery in chicken

Sample-Target (10^3 CFU/25 g)	Distance (depth)	Concentration of bacteria (CFU/total)	Concentration of bacteria (CFU/mL)	
		Resuspended second filter[1)]	Resuspended second filter	Estimated log reduction
Chicken- *Salmonella* Typhimurium	5 mm	57.3±14.3	28.7±7.1	2-log reduction
	1 mm	72.0±18.5	36.0±9.2	

1) Secondary filtrate obtained after double filtration of the inoculated sample using the FASP method.

2) Data were measured at least three times and are presented as mean±SD.

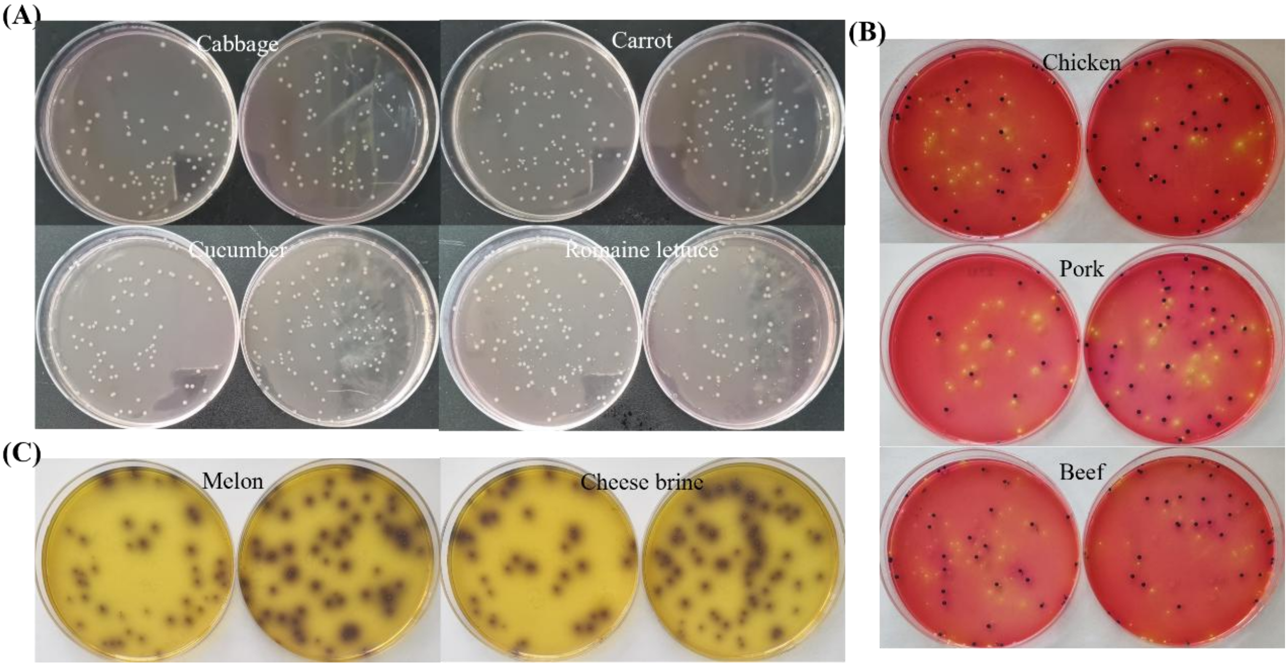

Figure S1. Plating results on selective media after filter-assisted sample preparation of real food samples. Selective media results for target bacteria in real food samples inoculated with 10^3 CFU per 25 g: (A) *Escherichia coli* O157:H7, (B) *Salmonella* Typhimurium, (C) *Listeria monocytogenes*.

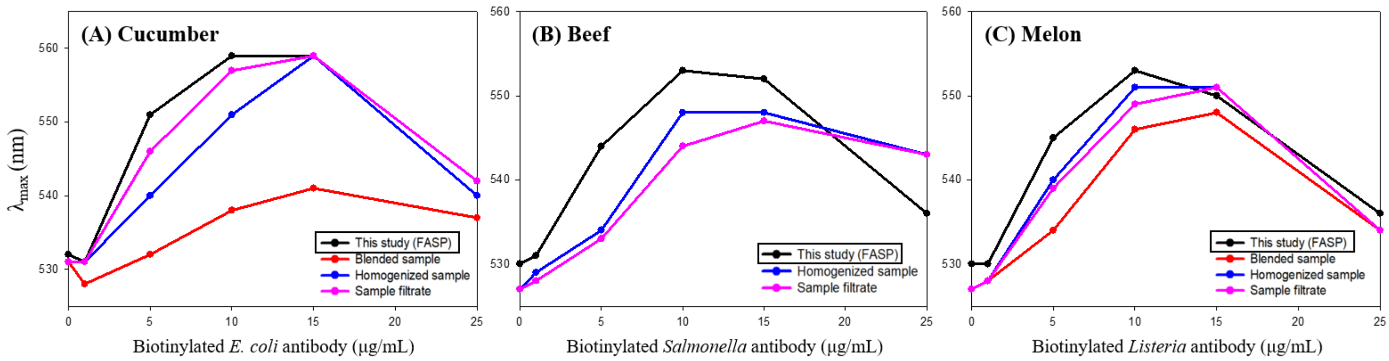

Figure S2. Changes in λ_{max} of colorimetric biosensors integrated with various pretreatment methods, as measured by spectrophotometry. (A) Cucumber, (B) beef, and (C) melon samples. From top to bottom: (i) sample treated with the method developed in this study, (ii) blended sample, (iii) homogenized sample, and (iv) sample filtrate. For the blended beef sample, excessive interference and color change hindered spectrophotometric measurements in the 400–800 nm range (data not shown). Only the pretreatment method developed in this study demonstrated detection stability across various food matrices, comparable to that observed in PBS buffer.

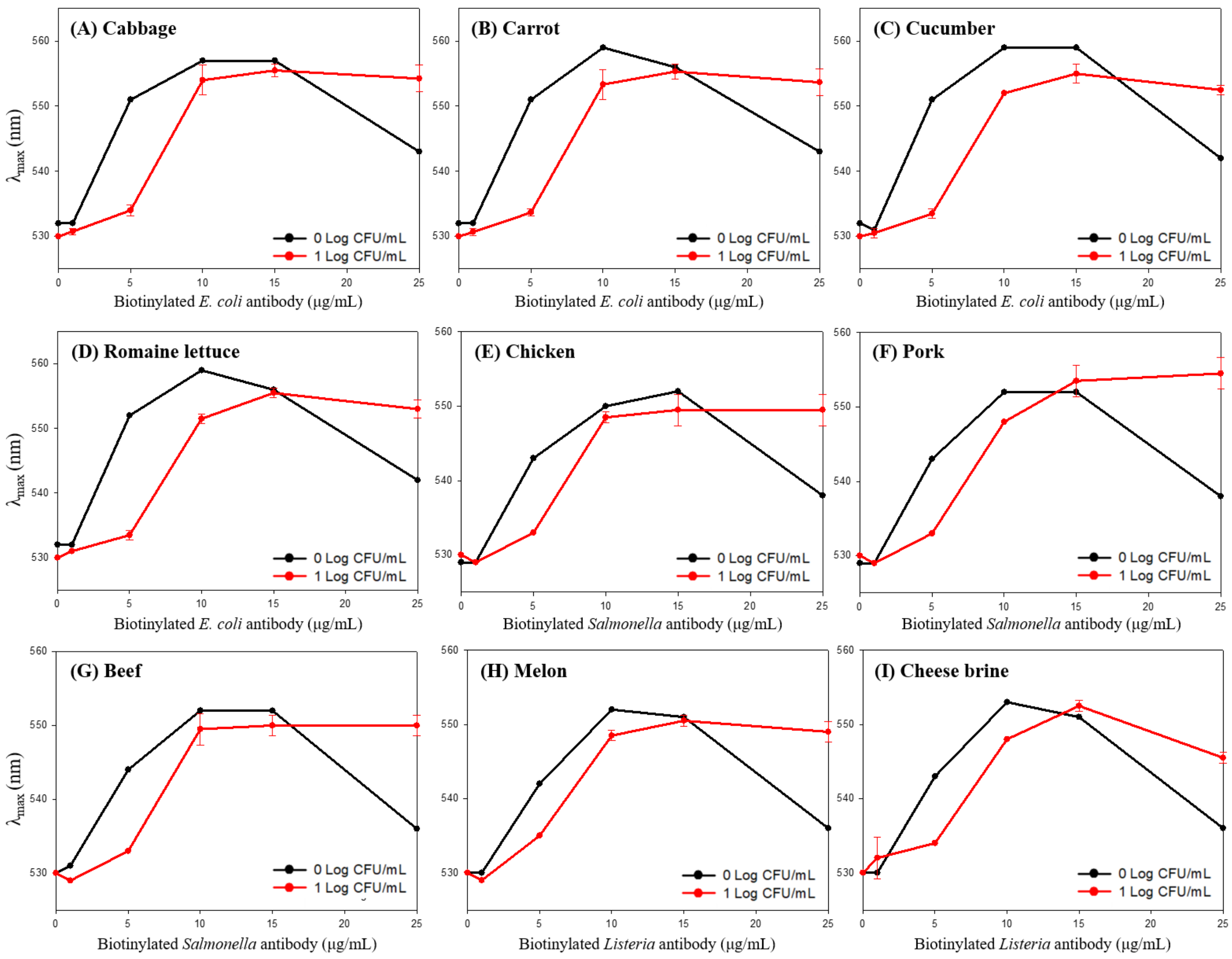

Figure S3. Variation in λ_{max} in response to foodborne pathogen detection in various food matrices using an immunoassay-based colorimetric biosensor. (A) Cabbage, (B) Carrot, (C) Cucumber, (D) Romaine lettuce, (E) Chicken, (F) Pork, (G) Beef, (H) Melon, (I) Cheese brine. Vegetable samples were inoculated with 10^2 CFU per 25 g, while meat, melon, and cheese brine samples were inoculated with 10^3 CFU per 25 g. All samples were subsequently processed using the integrated system developed in this study.
